# Emerging and Dynamic Biomedical Uses of Ferritin

**DOI:** 10.3390/ph11040124

**Published:** 2018-11-13

**Authors:** Brian Chiou, James R. Connor

**Affiliations:** Department of Neurosurgery, Penn State College of Medicine, Hershey, PA 17033, USA; bbc5138@psu.edu

**Keywords:** ferritin, iron, iron delivery, nanotechnology, nanocage, drug delivery, inflammation, serum biomarker

## Abstract

Ferritin, a ubiquitously expressed protein, has classically been considered the main iron cellular storage molecule in the body. Owing to the ferroxidase activity of the H-subunit and the nucleation ability of the L-subunit, ferritin can store a large amount of iron within its mineral core. However, recent evidence has demonstrated a range of abilities of ferritin that extends well beyond the scope of iron storage. This review aims to discuss novel functions and biomedical uses of ferritin in the processes of iron delivery, delivery of biologics such as chemotherapies and contrast agents, and the utility of ferritin as a biomarker in a number of neurological diseases.

## 1. Ferritin Introduction

Ferritin, a protein originally identified in 1937 by Vilém Laufberger [1], is a ubiquitously expressed iron storage protein most commonly characterized by its ability to accumulate and store up to 4500 atoms of iron [2]. Ferritin consists of 24 subunits, typically comprised of different ratios of the H and L chain subunit. The ratios vary by organ and even by cell type. Importantly, the different subunits have divergent functions—H-ferritin utilizes ferroxidase activity that is necessary for the oxidation of ferrous (Fe^2+^) to ferric (Fe^3+^) iron while L-ferritin contains acidic residues on the surface cavity of the protein that facilitate ferroxidase turnover and are crucial for the nucleation of ferric iron within the core of the fully formed protein. Historically, the primary function of the 24-mer protein has been to sequester extracellular and cytosolic iron and store this iron within its core for future use by the cell. This functionally prevents the formation of harmful reactive oxygen species (ROS) created through Fenton chemistry. Many reviews in the past few decades have focused on various aspects of the normal biological function of ferritin and its regulation pertaining to iron homeostasis [3,4,5,6]. In this review, we will outline novel and dynamic functions of ferritin that have recently come to light, specifically focusing on the novel uses for ferritin as an iron delivery protein, a delivery agent for numerous other biologics, and as a biomarker for various diseases.

Although traditionally characterized as a cytosolic protein, ferritin has also been found in mitochondria [7], plant plastids [8], the nucleus [9,10,11], and extracellularly in serum [12] and cerebrospinal fluid [13]. The different subcellular localizations of ferritin suggest a unique function for it in a variety of cell types. For example, nuclear ferritin reportedly binds to and protects DNA from UV-induced damage [9] as well as iron-induced damage [11]. The presence of ferritin in nuclei led to a series of studies by our group on the role of ferritin in cancer cells that revealed knockdown of ferritin increases sensitivity to radiation and chemotherapy in cancer cells [14]. Mitochondrial ferritin, while coded by a different gene than either L- or H-ferritin, shares 79% homology to H-ferritin and forms the classic ferritin shell [15]. In the mitochondria, free iron can cause potentially devastating effects; mitochondrial ferritin serves to sequester potentially harmful iron using ferroxidase activity [4].

## 2. Ferritin as an Iron Delivery Protein

**Figure 1:**
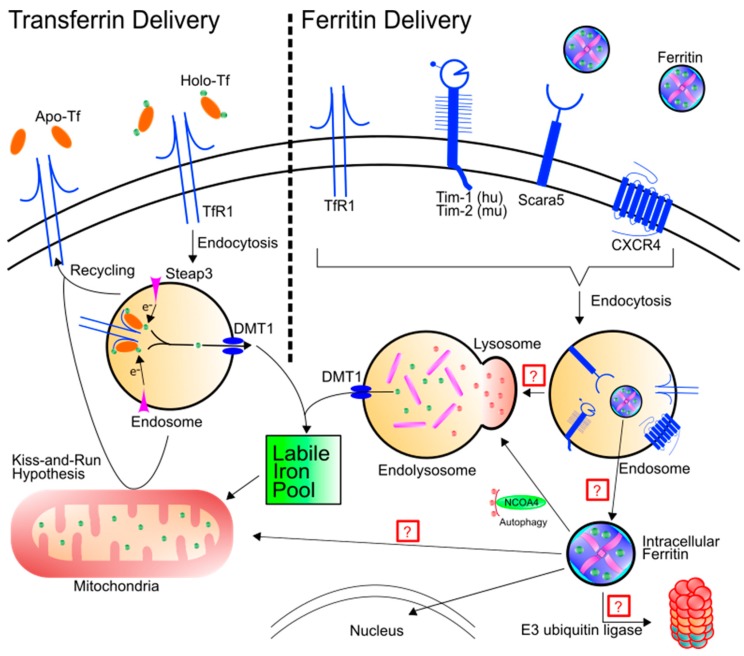
Schematic of transferrin and ferritin uptake and intracellular destiny. Left: Holo-Tf can bind to TfR1 and deliver iron into the labile iron pool through the endosome via DMT1. TfR1 can also be recycled to the cell surface, depositing apo-Tf into the extracellular matrix. An alternative hypothesis termed “kiss-and-run” results in endosomal delivery of iron to the mitochondria by brief interactions between the endosome and the mitochondria. Right: Ferritin binding has been shown for TfR1, Tim-1, Tim-2, Scara5, and CXCR4. After binding, ferritin is endocytosed. Note, while not all receptors are simultaneously required for endocytosis of ferritin, the schematic is demonstrating that all of the receptors in question have been found in the endosome. Ferritin trafficking after endocytosis has yet to be fully elucidated. Hypotheses include trafficking to the lysosome for protein degradation or for ferritin to leave the endosome through another means. Once ferritin is cytosolic, it can be shuttled to the lysosome via NCOA4 or it may be transported to the nucleus. There are a number of other potential hypotheses as well: (1) Ferritin may be poly-ubiquitinated and degraded by the proteasome or (2) ferritin may be shuttled to the mitochondria to deliver iron.

Iron delivery throughout the body is a vitally important process that is very tightly regulated. Iron deficiency may result in a number of longstanding and severe phenotypes such as anemia [16]. Similarly, iron overload, known as hemochromatosis, may result in organ failure [17] or significant inflammatory conditions [18]. Moreover, iron dyshomeostasis in the brain may result in significant neurodegeneration [19,20,21]. Excess organ and cellular accumulation in disease states occurs because the regulation of iron uptake is altered. The primary mechanism for iron uptake has historically been thought to be through the action of transferrin (Tf) delivering iron through transferrin receptor (TfR) (Figure 1). However, this paradigm may not address times of extreme iron need, such as during development and growth. While transferrin typically delivers two atoms of iron at a time, ferritin can potentially deliver a much more substantial amount of iron at a single time, allowing for rapid growth and iron utilization.

In particular, serum ferritin represents an important consideration in iron delivery; saturation of serum ferritin can range wildly depending on a number of factors, such as inflammation or development, with values ranging between 5% and 50% saturated [22]. It remains to be determined the extent to which serum ferritin functions to deliver iron; iron saturation of ferritin is largely ignored in the clinic. This is particularly important as around 90% of patients that present in the clinic with hyperferritinemia do not have iron overload [23]. While serum ferritin reportedly has a lower level of iron saturation than liver ferritin [24], the iron contained within serum ferritin was still a relatively higher amount than transferrin iron. Cohen et al. found that while liver ferritin contained 2074 atoms of iron per molecule of ferritin, serum ferritin contained 689 atoms of iron per molecule of ferritin [24]. Thus, despite the common tendency to refer to serum ferritin as “iron poor” that one consistently sees in the literature, it stands that if serum ferritin participates in iron delivery it is a significant source of iron.

**Figure 2:**
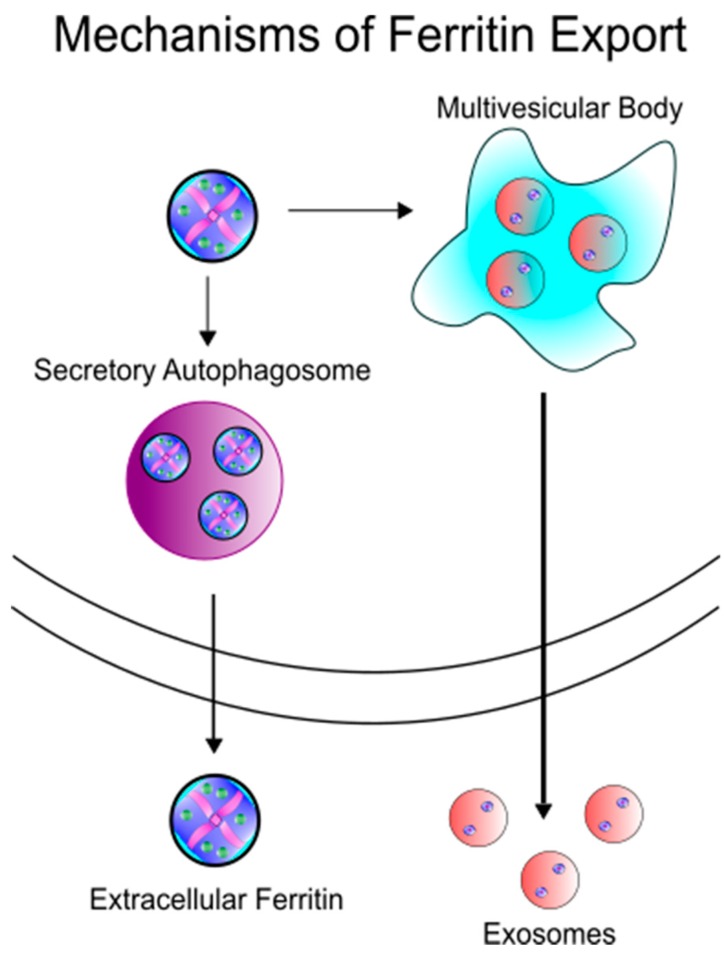
Schematic of iron export mechanisms. Ferritin has been hypothesized to be secreted in two different paradigms: (1) Ferritin is trafficked from the cell via secretory autophagy or (2) ferritin encapsulated in exosomes is released extracellularly from multivesicular bodies.

In order for ferritin to serve as an iron delivery protein, it must be released from cells (Figure 2). Secretion of ferritin has been demonstrated in many cell types, such as macrophages [18,25,26], hepatocytes [27], and Kupffer cells of the liver [28], in both healthy and disease settings. While the exact mechanism of ferritin secretion is still very much open for debate, the lack of glycosylation of extracellular ferritin suggests a non-classical secretion route. Cohen et al. have demonstrated the secretion of ferritin through the lysosomal secretory pathway, a cell-specific directed pathway that is not the product of damaged cells [24]. In this pathway, specialized secretory lysosomal granules are created both as degradative/modulatory compartments as well as storage areas, eventually followed by secretion of its contents to the extracellular milieu [29]. Subsequently Truman-Rosentsvit et al. found that in addition to the lysosomal secretory pathway, ferritin can also be secreted via the multivesicular body-exosome pathway [30]. Consistent with the secretion in membrane bound vesicles, Mrowcyznski et al. has demonstrated the presence of H-ferritin in exosomes [31]. Recently, Kimura et al. have further described this secretion mechanism as dependent upon the tripartite motif containing 16 (TRIM16) secretory autophagy receptor, avoiding autolysosomal degradation by pivoting ferritin cargo to the plasma membrane for secretion [32]. Thus, the data appear to support an active rather than passive release mechanism for ferritin from cells. Consequently, the amount of serum ferritin may be reflective of cellular processes and not a by-stander to inflammation and non-specific product of cell damage. This indicates that ferritin secretion may be an active response to physiological conditions and identifying those conditions that promote ferritin secretion are an area that could lead to new knowledge about ferritin function and iron requirements.

Recent studies have demonstrated ferritin ability for iron delivery. In particular, studies have provided evidence for H-ferritin uptake into the brain parenchyma [33], regulation of H-ferritin trafficking across the blood-brain barrier (BBB) [34,35,36,37,38], and H-ferritin uptake into the developing mouse brain (Unpublished Observations). In these experiments, radioactive ^59^Fe loaded into H-ferritin was used to track the transport of iron directly across the BBB in vitro [34] or directly into the brain in vivo [33]. This was further corroborated by inductively coupled plasma mass spectrometry (ICP-MS) studies of iron delivery by H-ferritin [35]. Todorich et al. demonstrate the importance of H-ferritin-mediated iron delivery to the brain, demonstrating that H-ferritin can replace transferrin as an obligate iron source for oligodendrocytes in vitro [39]. Further evidence has demonstrated both H- and L-ferritin binding and endocytosis in a number of different cell types, including hepatocytes [40], reticulocytes [41], lymphoid cells [42], and erythroid precursors [26,43,44]. In all of these cell types, binding and endocytosis of ferritin results in increased intracellular iron levels. The ability of ferritin to deliver a significantly higher amount of iron relative to transferrin suggests the existence of a more efficient iron delivery system perhaps especially in times of high iron demand, such as neural development and rapid growth [45].

There is considerable debate regarding the mechanism for ferritin uptake. While previous studies have demonstrated clathrin-dependent endocytosis of ferritin [46], there is not yet a consensus on the specific receptor mediating the uptake. Further complicating matters is the report that both H-ferritin and L-ferritin utilize different receptors. As previously stated, ferritin typically exists in a ratio of H- and L-ferritin; the ratio of H- to L-subunits may dictate the specific receptor preferred. While L-ferritin has been shown to bind to Scara5 [47], H-ferritin has thus far been shown to bind to Tim-2 in rodents [48,49,50], Tim-1 in humans [34,51], as well as CXCR4 [52] and TfR1 [53] (Figure 1). There is discrepant literature describing the ability for ferritin binding to saturate to Tim-2; one study found ferritin binding to be saturable [49] while another found it is not [50]. Importantly, we are not aware of any literature testing for respective contributions of ferritin binding between the TfR1 and Tim receptors. To this point, most cells in the studies studying TfR1 binding to ferritin did not assay for Tim-1/2 as a second uptake receptor. It is likely that both systems exist side-by-side and display different expressions for TfR1 and Tim-1/2 depending on cell type. This suggests that in cells that do not have TfR1, such as mature oligodendrocytes [54], ferritin internalization is dependent on Tim-1/2. Furthermore, it is well known that Tf receptors are saturated to almost 100% capacity when Tf saturation is normal (30%)—further suggesting that ferritin binding to Tim-1/2 may represent the preferred method for uptake of ferritin into the cell.

While binding and endocytosis of ferritin have been previously demonstrated, the eventual downstream fate of endocytosed ferritin has yet to be studied. An important consideration for ferritin uptake is that ferritin can transport anywhere from 100 to 1000 times as much iron as transferrin and therefore improper delivery and regulation of this delivery may have devastating consequences for the cell. As such, it is clearly critical to cell viability to have a route for handling iron release after exogenous ferritin uptake. For example, does ferritin get moved to the endosome and/or lysosome after uptake? If so, what happens to the iron contained within ferritin after ferritin degradation? Li et al. have previously demonstrated H-ferritin delivery to a human T lymphoblast cell line (MOLT-4), showing H-ferritin trafficking to the endosome as well as to the lysosome [53]. It remains to be seen if this delivery route is the same for all cell types, especially in cells lacking TfR1 such as mature myelinating oligodendrocytes [54]. It may also possible for ferritin to leave the endosome without being transported to the lysosome; the diameter of ferritin is only 12 nm, leading to a hypothesis that ferritin may leave the endosome through pores created under cellular stress [55] (Figure 1).

Previous characterization of the Tf-TfR axis has shown the ability for endosomal divalent metal transporter 1 (DMT1) to facilitate exit of iron from the endosome to be utilized by the cell [36,38]. Furthermore, previous studies have also demonstrated that H-ferritin transport is affected by pharmacological inhibition of DMT1 [34]. A second possibility for the fate of ferritin after uptake can be seen again in the Tf-TfR axis. Das et al. have shown that Tf may deliver iron to the mitochondria after uptake while still in the endosome, a process that has been termed “endosomal kiss-and-run” [56]. It is likely that this process, namely the delivery of extracellular iron to the mitochondria, is highly upregulated especially in times of high iron demand. Thus, the possibility exists for ferritin to perform a similar function as Tf to deliver a substantially higher amount of iron directly to the mitochondria either through “kiss-and-run” activity or through a directed transport mechanism (Figure 1).

Once ferritin is within the cytosol either via exogenous (uptake) or endogenous (translation) means, the fate of the protein itself and the iron contained within has been the subject of many studies, but the literature is inconsistent. Some studies suggest that intracellular ferritin is transported to the lysosome to be degraded and the iron inside the ferritin core to be recycled [27]. In particular, nuclear receptor coactivator 4 (NCOA4) was found to be essential in the transport of cytosolic ferritin to the lysosome, a process termed “ferritinophagy” [57]; NCOA4 knockout cells had reductions in ferritin degradation as well as lower intracellular labile iron [57,58]. These studies demonstrated that NCOA4 binds specifically to H-ferritin, but not L-ferritin, at the Arg23 residue [58]. Furthermore, NCOA4 is importantly recognized by the HECT-type E3 ubiquitin ligase HERC2 which itself is iron-sensitive, suggesting NCOA4 is degraded under conditions of iron excess. However, since NCOA4 only recognizes H-ferritin, there is still the question of how L-ferritin is degraded. Other literature suggests that ferritin in general is largely degraded in the proteasome and this degradation can be regulated based on proteasomal activity [59]. Still, others suggest that both lysosomal-mediated and proteasome-mediated degradation of cytosolic ferritin are indeed important contributors to overall ferritin homeostasis [60]. In 2010, Zhang et al. examined the degradation pathway by inhibiting either the lysosome or the proteasome and found that lysosomal proteolysis is the major contributor to ferritin degradation, with proteasomal-mediated degradation conferring a smaller proportion of the degradation [61]. Whether this finding is relevant to all cell types or only the mouse B cell line used in the Zhang et al. study is unclear.

## 3. Ferritin as a Delivery Agent

**Figure 3:**
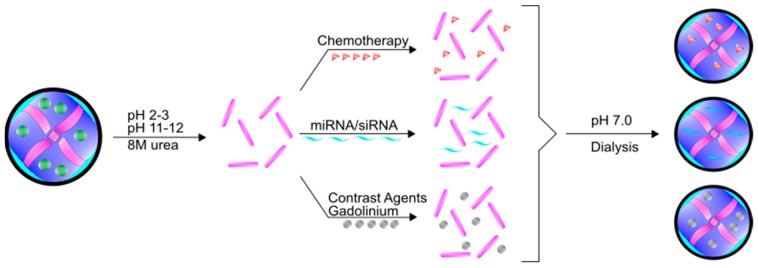
Schematic of encapsulation paradigms for ferritin. Fully formed 24-mer ferritin molecules can be disassembled using 8 M urea or acidic (pH 2–3) or basic (pH 11–12) conditions. A number of different molecules can be mixed with the disassembled ferritin, including chemotherapies, siRNAs/miRNAs, and contrast agents. Upon returning the ferritin mixture to neutral conditions (pH 7.0) coupled with dialysis, ferritin will spontaneously form the 24-mer around the molecule of interest. Free molecules can be dialyzed away from solution.

In addition to iron, ferritin has the unique ability to encapsulate and deliver other molecules. Indeed, ferritin is a highly preferred molecule in the field of nanotechnology owing to its self-assembly properties, precise cage alignment, and the ability to modify the surface of the fully formed protein with various conjugates to increase specificity and functionality. Importantly, despite the rigid structure under normal physiological conditions, ferritin can be disassembled when the pH becomes very acidic (pH 2–3) or very basic (pH 11–12) [62]. This process can be reversed by changing the pH back to neutral conditions, spontaneously forming ferritin into the full 24-mer [62] (Figure 3). Many researchers have utilized these assembly properties to load various compounds and molecules within the ferritin core. This flexibility in loading and assembly allows for numerous non-classical uses of ferritin. Moreover, even though ferritin has a high molecular weight (~474 kDa), the total diameter of the assembled 24-mer is roughly 12 nm. This diameter is easily smaller than many nanoparticles that are currently in development as drug delivery vehicles; the small diameter of ferritin permits wide movement across membranes, including into the nucleus and potentially out of endosomes [10]. Furthermore, while typically containing a negative surface charge, the charge on ferritin can vary depending on subunit composition, which allows for further flexibility when targeting ferritin molecules across different membranes [63]. Taken together, the unique characteristics of ferritin allow for robust nanocage assembly and targeted delivery.

One prominent area of investigation in the application of ferritin nanocages is in the treatment of cancer. Taking advantage of the fact that cancer cells require a higher amount of iron, many researchers have begun to use ferritin nanocages with encapsulated chemotherapies as delivery agents both in vitro [64,65] and in vivo [65,66] to great effect. Typically, chemotherapeutics such as doxorubicin, are modified by either forming a complex of the drug with transition metals such as Cu(II) [67] or by adding a charged accessory molecule such as poly-l-aspartic acid [68]. Not only does this complex facilitate entry into the ferritin core, but it also allows the transition metal or accessory molecule to stay associated with the ferritin protein while the drug is gradually released from ferritin [68]. For several chemotherapies that target the nucleus or DNA, the ability of ferritin to translocate into the nucleus represents a potential added benefit therapeutically [9,10]. Though the mechanism for drug release from ferritin either in the cytosol or in the nucleus has not yet been elucidated, there is strong evidence that delivery of chemotherapies via ferritin is an effective method that results in reduced non-specific cytotoxicity [69,70]. These reduced off-target effects suggest that cancer cells preferentially take up the ferritin nanocages in an attempt to obtain more iron from circulating ferritin. To further promote the uptake of ferritin by cancer cells, Li et al. conjugated epidermal growth factor (EGF) to H-ferritin as a way to target epidermal growth factor receptor (EGFR)-overexpressing breast cancers in mice [71].

While many studies of ferritin delivery of chemotherapies reveal strong efficacy in non-central nervous system (CNS) cancers [72,73,74], the use of ferritin nanocages is even more promising for therapeutics targeting brain cancers. Owing to the BBB, effective chemotherapeutic treatment of brain cancers has been extremely difficult. However, as previously described, new evidence suggests ferritin can traffic across the human BBB through Tim-1 [34], potentially allowing for the delivery of directed therapeutics into the brain parenchyma. In diseases such as neuroblastoma or glioblastoma that are extremely aggressive and have a severe phenotype with limited treatment options, ferritin nanocages represent a highly exciting and novel delivery route (Figure 2). As an example, Chen et al. have recently demonstrated that apoferritin loaded with doxorubicin can reliably cross the BBB and deposit the chemotherapy specifically within the tumor in a mouse model resulting in increased survival [75]. This example again demonstrates the preferential uptake of ferritin by cancer cells, likely in an attempt to obtain more iron.

In addition to loading the ferritin molecule with chemotherapies such as doxorubicin, the ferritin molecule may allow for delivery of small interfering RNAs (siRNAs) in a number of different biological paradigms. For example, Li et al. have demonstrated a simple method for encapsulating siRNA and using this ferritin-siRNA combination to perform high efficiency transfections of human primary mesenchymal stem cells (hMSCs) and peripheral blood mononuclear cells (PBMCs) in vitro when compared to commercially available Lipofectamine [70]. This study demonstrated that ferritin-siRNA could achieve high gene expression silencing with a low siRNA concentration of 10 nM. This work has large implications in the treatment of various immune-related disorders, as in vivo delivery of this ferritin-siRNA complex did not show any immune activation of the PBMCs but was preferentially taken up by activated B- and T-cells. Furthermore, it has been shown that delivery of H-ferritin siRNA sensitizes glioma to radiation [14,76] which brings up the possibility of using ferritin containing H-ferritin siRNA in order to sensitize various cancers to radiation.

In 2013, Kanekiyo et al. detailed an exciting use for ferritin as a natural delivery agent for vaccines [77]. In this study, the authors fused the influenza virus haemagglutinin (HA) to the ferritin protein and immunized mice with this fusion protein. As a result, mice produced broad-scale neutralizing antibodies against common and vulnerable targets of universal influenza vaccines. Furthermore, the HA-ferritin fusion protein elicited ten times as high of a response as the naïve vaccine, demonstrating increased potency and breadth of influenza immunity. Kanekiyo and colleagues have also extended this approach to the creation of a potential vaccine for Epstein-Barr Virus [78]. This seminal study spawned other studies such as one by Han and colleagues—priming of dendritic cells using ferritin nanocages conjugated to ovalbumin antigenic peptides OT-1 and OT-2 [79]. In this study, the dendritic cell-based vaccine was developed by putting OT-1 and OT-2 on the exterior surface and interior cavity, respectively, resulting in effective priming of CD4^+^ and CD8^+^ T cells after antigen presentation by the dendritic cell. Immunization of mice with this vaccine resulted in differentiation and proliferation of CD4^+^ and CD8^+^ T cells specifically targeting OT-1 and OT-2.

## 4. Ferritin as a Mineralization Chamber

Magnetic resonance imaging (MRI) is a powerful diagnostic technique with extensive uses in the detection of tumor development [80], white matter development and injury [81,82], and aberrant vascularization [83,84]. To create a clinically useful image that is both highly sensitive and accurate, contrast agents are typically used to aid the visual interpretation of MRI scans. The most common contrast agents used are different formulations of gadolinium (Gd), owing to the strong paramagnetism of Gd from seven unpaired electrons. However, a significant problem with Gd is that it nonspecifically labels all highly vascularized tissues, resulting in false positives and a loss of spatial resolution.

Ferritin represents a viable alternative to Gd labeling for MRI. Both endogenous and exogenous ferritin have found great utility in improving MRI contrast capabilities and disease monitoring [85]. In particular, an increased amount of iron within the ferritin core directly correlates with T2* contrast power. It has been shown that Gd can also be loaded within the ferritin core and used as a more prominent imaging agent [72,86] (Figure 2). This function of improved contrast has many applications, especially in the diagnosis or monitoring of cancer progression. Other functionalities of ferritin use in MRI rely on preferential uptake of ferritin by resident macrophages within a cancer tumor [73] or by the tumor cells themselves either by native uptake or by enhancing uptake with further conjugations [87].

An interesting use of ferritin on an industrial process-scale level is the use of thermostable ferritin from *Pyrococcus furiosus* as a water treatment biologic [88]. In this example, ferritin is used to sequester excess phosphate and arsenate due to the high phosphate and arsenate content of seawater and industry waste. While current phosphate removal methods use a system that is heavily reliant on calcium and aluminum sulfates or bacteria that adsorb calcium, the removal of phosphates by ferritin represents a lower maintenance cost and a method that can be recycled [88].

## 5. Ferritin as a Marker for Inflammation

There are three main regulatory pathways for the expression of ferritin: The iron response proteins (IRPs)/iron responsive element (IRE) system [89], the regulation of transcription through NF-κB [90], and transcriptional regulation through the hypoxia inducible factor 1 alpha (HIF-1α) binding to a HIF responsive element (HRE) upstream of the IRE system [91]. The IRP/IRE system functions by the binding of IRPs to the IRE at the 5’ untranslated region (UTR) of ferritin mRNA under conditions of low iron, inhibiting its translation. However, in conditions of high iron, IRP binding to the IRE is decreased, leading to increased ferritin expression. Similarly, ferritin gene transcription is upregulated in conditions of inflammation where inflammatory cytokines such as tumor necrosis factor alpha (TNF-α) and interleukin-2 (IL-2) signal to increase binding of NF-κB to the transcription enhancer FER2 upstream of the IRE and coding region [90]. It has been shown that the NF-κB pathway is highly responsive to inflammation [92]. Evidence for this pathway stems from activation of toll-like receptor 2 (TLR2) in macrophages resulting in IRP-independent upregulation of H-ferritin, as well as direct pharmacological activation of the NF-κB pathway [93]. Similarly, it has been shown that H-ferritin expression is also responsive to tumor necrosis factor alpha (TNF-α) [94], interleukin-2 (IL-2) [95], and IL-10 [96]. Consequently, the elevated presence of ferritin in serum has long been considered in clinical settings to be an acute phase inflammatory marker, as elevated serum ferritin levels have been correlated with increased levels of pro-inflammatory cytokines [97,98]. Potential sources of serum ferritin during inflammation include secretion by macrophages [24,25] and/or release from cells due to tissue damage [99], both indicators of inflammation or infection. Importantly, there is disagreement in the literature about whether serum ferritin is a pro-inflammatory marker [100], an anti-inflammatory marker [101,102], or is simply a byproduct of release during the course of infection by damaged cells [99]. Understanding mechanisms of release of ferritin and how those mechanisms are influenced at the cellular level could provide insights into the biological and clinical meaning of ferritin in serum.

Serum ferritin has also been routinely interpreted in the clinic as a surrogate measure of iron storage in the body [103]. There are numerous studies that examine the correlation between serum ferritin and iron deficiency anemia or hemochromatosis in which serum ferritin levels are decreased or elevated, respectively [104]. However, inflammation or infection is known to drastically change the amount of serum ferritin present [105]. Accordingly, in an individual with acute or chronic inflammation, the extent to which serum ferritin levels reflect inflammation may reflect the total body iron stores. To further complicate this understanding, there are studies demonstrating that serum ferritin is not the most reliable marker for total body iron stores, especially when there are other complicating factors such as liver or kidney damage [12]. Put together, it seems that clinically, serum ferritin may be used as a marker for total body iron stores in the healthy individual, however, in a disease setting serum ferritin may be more indicative of the underlying pathophysiology such as organ damage or infection.

Another important consideration is while serum ferritin may be used clinically to suggest inflammation, serum ferritin measurements are focused largely on the L-ferritin subunit and as such, remain a poor indicator of iron status of the patient in this inflammatory state [97], as described earlier it is H-ferritin that is mostly responsive to the inflammatory process. H-ferritin has been detected in serum [106] and has the potential to be much more dynamic and informative than L-ferritin. A critical question that has yet to be answered clinically is: Is the iron saturation of serum ferritin in any given individual more informative to the total body iron stores than serum ferritin levels itself? Currently, it is rather trivial to measure serum ferritin levels but to measure the iron contained within serum ferritin is considerably more difficult. One recent study has looked at ferritin secretion from macrophages and determined that iron saturation of the ferritin was about a third as saturated as liver ferritin [24]. This is important as many consider serum ferritin to be iron-free or iron-poor, but the reality is that it contains a significant amount of iron. Thus, ferritin saturation may be the better measure of total body iron stores rather than the serum ferritin molecules themselves.

In addition to the utility of serum ferritin clinically, intracellular ferritin has also been implicated in the host response to microbial infection. Recent evidence demonstrates that H-ferritin confers tolerance to malaria and sepsis during infection by limiting reactive oxygen species (ROS) and overall oxidative stress. For example, Gozzelino et al. shows evidence that the activity of H-ferritin confers a metabolic adaptation to *Plasmodium* infection [107]. This activity is through the chelation ability of H-ferritin to limit labile iron from activating the pro-apoptotic c-Jun N-terminal kinase (JNK). Furthermore, the authors found that JNK activity inhibits H-ferritin expression and causes increased susceptibility of malaria coupled with tissue damage and oxidative stress. To extend this study, Weis et al. also looked at this metabolic adaptation in sepsis, a devastating syndrome resulting from a maladaptive host response to infection [108]. Much in the same way as Gozzelino et al., Weis and colleagues demonstrated a disease tolerance to sepsis that was facilitated by H-ferritin. Importantly, activity of H-ferritin to sequester cytosolic iron was critical for the setup of disease tolerance. Weis et al. showed that H-ferritin iron chelation prevents iron-driven oxidative inhibition of the glucose-6-phosphatase to sustain glucose production via liver gluconeogenesis. Another recent study that demonstrates the importance of H-ferritin in response to microbial infection was by Reddy et al. [109]. In this study, the authors infected H-ferritin knockout mice or wild-type mice with *Mycobacterium tuberculosis*, demonstrating that knocking out H-ferritin specifically in myeloid cell populations decreased survival after infection, increased the overall inflammatory response, and increased the total bacterial loads. These studies highlight the general importance of ferritin in the inflammatory response as well as for normal host iron homeostasis.

In contrast, ferritin has also been shown to have a role as an anti-inflammatory molecule. Ferritin in serum reportedly binds to high molecular weight kininogen (HK) [110]. Normally, HK is a co-factor in the intrinsic coagulation pathway; HK cleavage by the serine protease kallikrein leads to release of bradykinin and two-chain high molecular weight kininogen [111]. This particular pathway has large implications in the progression of a number of inflammatory diseases such as asthma [112], inflammatory bowel disease [113], and rheumatoid disorders [114,115]. Ferritin binding to HK serves as an anti-inflammatory signaling pathway, as the direct interaction of ferritin with HK prevents HK cleavage and a concomitant reduction in bradykinin release [102]. This overall interaction diminishes the total inflammatory response. In a similar paradigm, Fan and colleagues demonstrated that L-ferritin can play an important anti-inflammatory role in lipopolysaccharide (LPS)-stimulated macrophages [101]. In this study, Fan et al. show that overexpression of L-ferritin in macrophages can inhibit LPS-mediated transcription of TNF-α and IL-1β, both critical pro-inflammatory cytokines. Conversely, they showed that L-ferritin is significantly downregulated after LPS stimulation. From this study, the authors conclude that L-ferritin has an underappreciated role in the anti-inflammatory resolution cascade. Importantly, both of these studies are direct evidence of ferritin playing an anti-inflammatory role.

## 6. Ferritin as a Disease Biomarker

While the amount of iron has been shown to increase throughout the body with age [116,117,118], abnormal iron homeostasis can lead to devastating diseases. Typically, a concomitant increase in ferritin levels are paired with increased iron levels [119], attempting to ensure safe handling of this increase in iron. However, a failure of ferritin levels to concomitantly increase with iron may impact normal physiology and induce pathology as a result of iron-mediated induction of oxidative stress. This role for ferritin and a mishandling of systemic iron has been shown to be implicated in a number of diseases such as Still’s disease, hemophagocytic syndrome, and sideroblastic anemia—implications of serum ferritin in these diseases and others have been critically reviewed in numerous other reviews [12,104,106]. Similarly, serum ferritin as a bioindicator for cancer has been extensively reviewed as well [120,121]. Here we will focus specifically on neurological disorders and the impact that ferritin has on diagnosis as a biomarker for disease or progression. One interesting new development has been proposed in Friedreich’s Ataxia where the authors discuss the hypothesis that the mitochondrial protein frataxin may oligomerize like ferritin and perform functions redundant with mitochondrial ferritin, acting as another iron storage molecule [122]. Loss of frataxin and this iron storage property may result in Friedreich’s Ataxia and subsequent neurodegeneration.

### 6.1. Neuroferritinopathy

Neuroferritinopathy (NF), an autosomal dominant disease resulting from mutations in L-ferritin, is classified as belonging to a group of movement disorders known as Neurodegeneration with Brain Iron Accumulation (NBIA). This disease is an extrapyramidal syndrome typically characterized by excessive iron accumulation in the basal ganglia. NF was originally characterized as a disease arising from an additional adenine at c.460 of exon 4 in the L-ferritin gene, but other mutations around the same region were quickly identified as contributing to disease pathology [123]. These mutations cause a frameshift in the C-terminus of the L-ferritin subunit resulting in reduced or ablated iron storage and subsequent iron-mediated damage, as this mutation creates a larger than normal pore [123]. In 2010, Barbeito et al. showed that fibroblasts from NF patients have higher levels of H- and L-ferritin as well as increased levels of total iron stores [124]. Furthermore, Maccarinelli and colleagues showed in 2014 that mice with the same L-ferritin mutation had a progressive impairment in motor coordination and a propensity to accumulate iron within post-natal hippocampal neurons [125]. Interestingly, this mutation in NF patients is present in all cells, but there is no evidence for iron mismanagement or dyshomeostasis by the rest of the body. It is currently unclear whether brain cells are simply more sensitive to iron-dependent toxicity or if there is a separate function for L-ferritin within the brain parenchyma. It is likely that the high oxygen and glucose consumption of the brain leads to an increase in iron utilization and therefore ROS/oxidative stress. Without functional L-ferritin, brain cells are rendered unable to survive this oxidative damage.

### 6.2. Parkinson’s Disease

One of the most prominent neurodegenerative diseases with marked neurodegeneration as well as substantial iron accumulation in the substantia nigra pars compacta (SNpc) is Parkinson’s disease (PD). In general, there is agreement that iron accumulation in the SNpc increases with age, but the additional increase in iron in the SNpc as a result of PD depends heavily on the stage and progression of PD [126]. With regard to ferritin in PD, there are a number of conflicting reports regarding the expression of ferritin within the SNpc; biochemical studies have shown both increased [126] and decreased [127] levels of ferritin within the SNpc in PD. In the 1-methyl-4-phenyl-1,2,3,6-tetrahydropyridine (MPTP) model of PD, H-ferritin overexpression in neurons is protective against disease progression [128]. Although there seems to be altered iron management specifically within the SNpc, cerebrospinal fluid (CSF) levels of ferritin and serum ferritin levels were not significantly different between PD and non-PD patients [127]. In contrast however, there is a recent study detailing how serum ferritin may serve as a potential biomarker for diagnosis in PD [129]. In this study, the authors found an increased level of serum ferritin in PD patients when compared to non-PD patients. Unfortunately, the sample size of the patient cohort used in this study was relatively small (40 healthy vs. 40 PD patients) and was limited to a cross-sectional analysis. Furthermore, there is a lack of consensus about the reliability of using serum ferritin as an indicator of brain iron status or even, as previously discussed, systemic iron status. Another consideration in the utilization of serum/CSF ferritin as a biomarker in PD is that peripheral inflammation plays a large role in PD disease progression [130]. As such, serum/CSF ferritin levels will likely be altered as a result and may not be informative of brain iron status.

### 6.3. Restless Legs Syndrome

In Restless Legs Syndrome (RLS), a disorder characterized by an irresistible urge to move the limbs and unpleasant sensations in the legs, a well-established neurobiological abnormality is low brain iron despite normal peripheral iron levels [131]. However, RLS is about five times more prevalent in iron deficient populations [132,133]. It is clear that ferritin levels are significantly impacted by and are important in RLS pathology, but it is not yet known how ferritin may specifically contribute to disease progression or disease symptoms. We have previously shown that Tf, TfR, and H-ferritin levels are decreased in the endothelial cells of RLS patients, indicating iron mismanagement at the level of the BBB, suggesting decreased brain iron acquisition in RLS brains [134]. Furthermore, studies have also demonstrated a decrease in H-ferritin and iron levels specifically in the SNpc of RLS brains compared to controls [135]. Currently, serum ferritin is used to guide treatment, but we have discussed the concerns regarding the utility of serum ferritin both as a measure of total body iron stores and serum ferritin levels are of no value in predicting response to treatment. Lammers et al. studied serum ferritin, bodily iron, and RLS, finding no association between early-onset RLS and longitudinal measures of iron stores [136]. Similarly, while serum ferritin is currently used clinically to guide treatment, it has been shown that serum ferritin levels have no predictive value in the response to treatment [131]. This is an important consideration as it underlines the fact that serum ferritin levels are not necessarily predictive of brain ferritin levels or total brain iron status.

### 6.4. Amyotrophic Lateral Sclerosis

Amyotrophic Lateral Sclerosis (ALS), a progressive and devastating disease, is characterized by loss of motor neurons in the cortex, brainstem, and spinal cord. While ALS is a relatively common disease, the exact mechanisms for this neurodegeneration is currently unknown, with a number of factors such as oxidative stress, excitotoxic stimulation, and genetic factors all implicated to play a role. It has long been known that in the ALS iron homeostasis is disrupted; there is higher iron in the CSF and spinal cord of ALS patients [137]. While there are a large number of studies that demonstrate elevated levels of serum ferritin in ALS [137,138,139], there is not yet a consensus regarding whether serum ferritin is predictive [139,140,141] or unrelated [137,138] to disease progression and survival rates.

## 7. Conclusions

Classically, ferritin has been described as a critical iron storage protein whose dual roles as both an antioxidant and an iron sink were essential to cellular survival. The importance of ferritin can directly be seen in knockout mouse models; both H and L homozygous knockout mice are embryonic lethal [128,142]. While not disputing the importance of these intracellular roles, it is clear that ferritin has highly dynamic and novel functions outside of the cells. Recent evidence has demonstrated the ability of ferritin to participate in iron delivery, delivery of macro/micromolecules, and as a potential biomarker in many neurologic diseases. Though serum ferritin is largely comprised of the L subunit, iron levels in ferritin remain much higher than transferrin; there is significant potential for serum ferritin to deliver anywhere between 10 to 100 times higher iron than transferrin [35]. Furthermore, while it is clear that ferritin can deliver iron to various organs throughout the body, it is still unclear as to why the body needs multiple iron delivery routes, especially with such drastic differences in amount of iron delivered. Besides iron, the delivery of ferritin-encapsulated macro- and micromolecules has important implications in the treatment and diagnoses of various disorders. While serum ferritin has been implicated in a number of diseases, the exact role and mechanism for serum ferritin in these disorders has not yet been fully characterized. There is a large volume of literature dedicated to exploring ferritin biology, but a number of questions and topics remain to be explored regarding ferritin delivery, downstream fate, role of ferritin in many diseases, and the utility of ferritin clinically.
